# Biochar amendment immobilizes lead in rice paddy soils and reduces its phytoavailability

**DOI:** 10.1038/srep31616

**Published:** 2016-08-17

**Authors:** Honghong Li, Yuting Liu, Yanhui Chen, Shanli Wang, Mingkuang Wang, Tuanhui Xie, Guo Wang

**Affiliations:** 1 College of Resource and Environmental Science, Key Laboratory of Soil Environmental Health and Regulation in Fujian Province, Fujian Agriculture and Forestry University, Fuzhou 350002, PR China; 2 Department of Soil and Environmental Sciences, National Chung-Hsing University, Taichung, 10402, Taiwan; 3 Department of Agricultural Chemistry, National Taiwan University, Taipei, 10617, Taiwan

## Abstract

This study aimed to determine effects of rice straw biochar on Pb sequestration in a soil-rice system. Pot experiments were conducted with rice plants in Pb-contaminated paddy soils that had been amended with 0, 2.5, and 5% (w/w) biochar. Compared to the control treatment, amendment with 5% biochar resulted in 54 and 94% decreases in the acid soluble and CaCl_2_-extractable Pb, respectively, in soils containing rice plants at the maturity stage. The amount of Fe-plaque on root surfaces and the Pb concentrations of the Fe-plaque were also reduced in biochar amended soils. Furthermore, lead species in rice roots were determined using Pb L_3_-edge X-ray absorption near edge structure (XANES), and although Pb-ferrihydrite complexes dominated Pb inventories, increasing amounts of organic complexes like Pb-pectins and Pb-cysteine were found in roots from the 5% biochar treatments. Such organic complexes might impede Pb translocation from root to shoot and subsequently reduce Pb accumulation in rice with biochar amendment.

Lead (Pb) is a toxic element commonly found in heavy-metal contaminated soils, and it has been one of the major global environmental concerns over the past few decades, especially in developing countries^1^. Plants play important roles in trapping and removing Pb from contaminated soils and affect its global transport. However, when Pb is excessively accumulated in the edible parts of crops, it can cause harm to human beings who consume the crop. For example, Pb can accumulate in the brain and severely affect the development of intelligence in children^2^.

Rice is widely cultivated globally, especially in southeastern Asia, and is a staple food for many people. It has been shown that rice accumulates considerable amounts of heavy metals in its grains^3^,^4^; therefore rice is assumed to be an important source of dietary heavy metal intake for people who consume it regularly. Indeed, studies have confirmed that Pb pollution in rice grains is common in some regions of China, especially in areas where the soil has been heavily polluted with Pb^5^.

Immobilization with amendments is a practical and low-cost measure that can be used to inhibit the uptake of heavy metals by crops grown on contaminated agricultural soils, especially in the countries or regions where there is a shortage of arable land. Immobilizing agents, such as Ca-, P-, and Si-containing materials, can selectively reduce the availability or mobility of heavy metals in the soil by adsorbing, complexing or precipitating heavy metals^6^.

In contrast, biochar is a solid carbonaceous residue produced by burning biomass under oxygen-free to oxygen-deficient conditions, and is increasingly being used to immobilize heavy metals in soils. Immobilization with biochar is considered as a promising method, owing to its distinct physicochemical properties, such as a surface area that is rich in organic groups, a alkaline pH, and a high carbon: nitrogen (C:N) ratio^7^,^8^. Biochar made from various raw materials has already been shown to effectively reduce the availability and mobility of heavy metals, such as Cd, Pb, Cu, and Ni, in soils^9^,^10^,^11^. And both Extended x-ray absorption fine structure (EXAFS) spectroscopy and chemical extractions have shown that, when soils are amended with biochar, Pb is transformed into more stable Pb-hydroxide, chloropyromorphite and Pb-phosphate^12^,^13^. Moreover, immobilization may also reduce Pb concentrations in the edible parts of crops^14^.

However, the inhibitory effect of an amendment on the accumulation of Pb in a soil-plant system depends not only on a decrease in the available Pb in soil, but also on a decrease in the translocation of Pb within the plants. Many studies have demonstrated that biochar reduces both the available Pb in soil and the Pb concentration in the edible parts of crops^9^,^11^,^12^,^15^, although not unanimously^14^. A few studies have also focused on the transfer characteristics of Pb accumulation in rice grains and revealed potential mechanisms underlying Pb retention in different parts of rice after soil amendment with biochar.

The transformation of Pb species in paddy soils and in different rice tissues after biochar applications remains unclear. Therefore, the objectives of this study were to investigate: (1) the Pb immobilization mechanisms in paddy soils and rice plants, and (2) the relationship between Pb accumulation in rice grains and the Pb transfer factors between different parts of the soil-rice system after biochar application.

## Results and Discussion

### Effects of biochar on Pb availability in the soil

Significant (One-way analysis of variance (ANOVA), *P* < 0.05) increases of 86.9% of water-soluble organic carbon (WSOC) and soil pH increased from 5.63 to 6.81 during tillering stage were found with the 5% biochar amendment, but the values of WSOC and soil pH at maturity stage were lower than tillering stage (Table 1). The biochar produced at a pyrolytic temperature of 500 °C had exhibited a higher pH, and the pH increase is due to the release of alkali salts from the feedstock during pyrolysis^16^. Then, because higher pH favors the dissolution of soil organic matter^17^, increased WSOC was also observed.

The CaCl_2_− and diethylene triamine pentaacetic acid (DTPA)-extractable Pb were significantly (*P* < 0.05) reduced in soils with biochar amendments at tillering and maturity stages (Table 1). Available soil Pb decreased more significantly with increasing biochar amendments. With 5% biochar amendment, CaCl_2_-extractable Pb decreased 96.7%, comparing with control (in absence biochar), at the tillering stage and 94.3% at the maturity stage, whereas DTPA-extractable Pb decreased 17.5% at the tillering stage, and 16.6% at the maturity stage. These results agree with a previous study, which found that CaCl_2_-extractable Pb declined sharply with the addition of biochar, whereas DTPA-extractable Pb fell slowly^14^. In addition, we also found that CaCl_2_- and DTPA-extractable Pb increased from the tillering stage to the maturity stage, which could be attributable to the decrease in soil pH.

**Figure 1:**
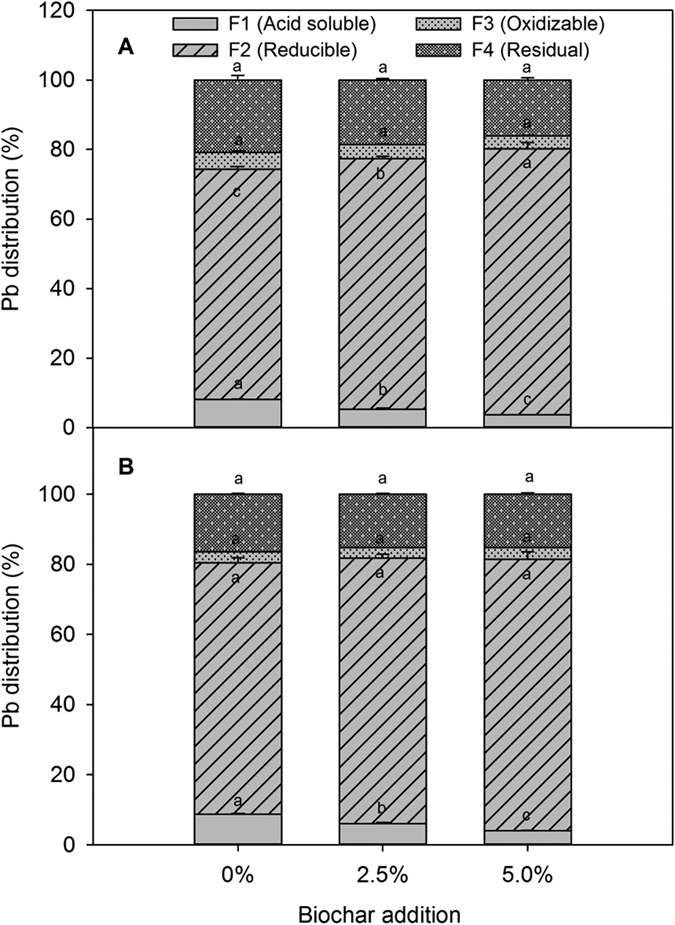
Lead percentage (%) showing the European Community Bureau of Reference sequential fractionations of soils at the tillering (**A**) and the maturity stages (**B**).

The mobility and availability of Pb in soils are largely controlled by the chemical forms of Pb present and decrease in the following order: acid soluble forms >reducible forms >oxidizable forms >residual forms^18^. In our study, when 5% biochar was added, acid soluble Pb (II) was reduced significantly (*P* < 0.05) from 8.12% (control) to 3.67% at the tillering stage, and from 8.75% (control) to 4.03% at the maturity stage. In contrast, reducible Pb increased by 10.35 and 6.27% at the tillering and maturity stages, respectively (Fig. 1). These results suggested that after biochar application, acid soluble Pb (II) was partly transformed into reducible Pb in the soils, which agreed with a previous study that showed that Pb was immobilized by the formation of surface complexes between Pb^2+^ and functional groups on biochar when rice straw derived biochar was added to a polluted Ultisols^16^.

Soil pH was considered a critical factor in determining Pb availability, and in our study, we found that CaCl_2_-extractable Pb was negatively correlated with pH (r_tillering stage_ = 0.78, *P* = 0.012; r_maturity stage_ = 0.74, *P* = 0.022; n = 9). This relationship was agreed with previous results that indicated that Pb phytoavailability decreases as the pH increases^19^. As pH increases, the hydrolysis of Pb is also increased, leading to more specific adsorption of Pb by the variable charge of the soils and more precipitation with phosphate as pyromorphite^13^,^16^. In addition, increased pH could cause an increase in Si concentration in pore water and silicon accumulation in rice plant resulted in Pb toxicity alleviation by some physiological mechanisms^20^,^21^. Lead can also be immobilized *via* specific adsorption onto the biochar particles due to the increased density of cation-exchange sites on the biochar surfaces^22^.

### Effects of biochar on the Fe and Pb concentrations in iron plaque

**Figure 2:**
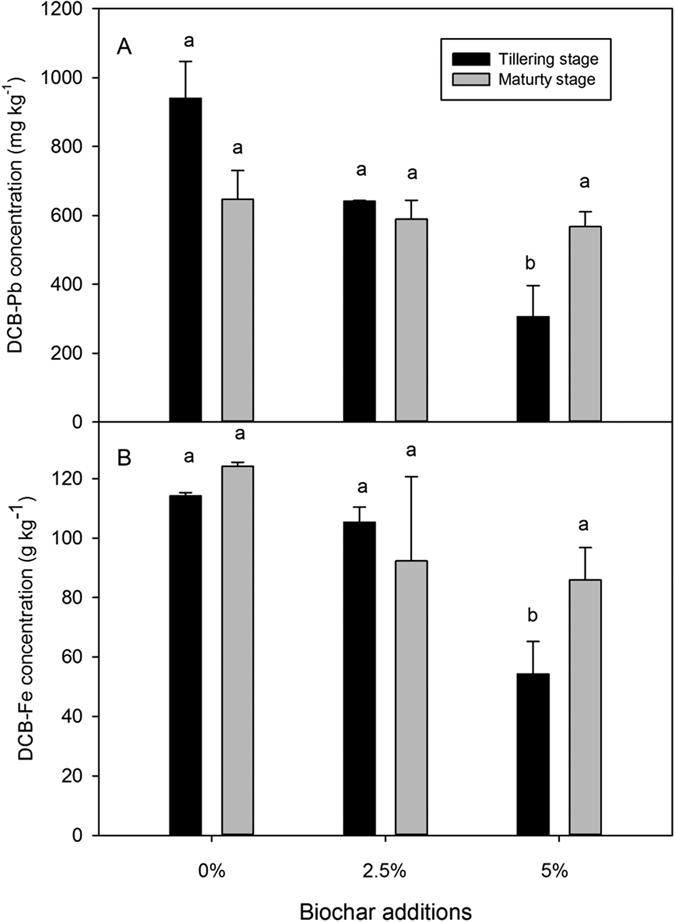
Concentrations of dithionite-citrate-bicarbonate (DCB)-extractable Pb (**A**) and Fe (**B**) (g kg^−1^) in iron plaques as a function of biochar amendment level.

Figure 2 showed the amounts of iron plaque (denoted as dithionite-citrate-bicarbonate (DCB)-extractable Fe) on the root surfaces after the different biochar amendments. With increasing biochar amendments, the DCB-extractable Fe (DCB-Fe) of roots was reduced significantly (one-way ANOVA, *P* < 0.05) at both tillering and maturity stages, and a similar trend was observed for DCB-extractable Pb (DCB-Pb). The formation of iron plaque is affected by many factors, including soil properties, root oxidation, and Fe (II) concentration in the soil rhizosphere. Therefore, it is likely that higher proportions of biochar resulted in a higher soil pH and lower water-soluble Fe in the soil solution, and ultimately, resulted in reduced Fe-plaque formation.

Formation of Fe-plaque is also related to plant growth stage. DCB-Fe decreased from the tillering to the maturity stage. However, the Pb concentration in the roots (Fe-plaque removed) increased from the tillering to the maturity stage (Table 2). Generally, Fe-plaque has a high adsorption capacity for heavy metals due to its iron hydroxide functional groups^23^,^24^, and although it has also been reported that Fe-plaque fails to affect heavy metal uptake by plants^24^, prior research^25^ demonstrated that the increased capacity of Fe-plaque to retain Pb is the main mechanism for reducing Pb accumulation in rice shoots.

### Effects of biochar on the concentration of Pb in different rice plant tissues

The Pb concentrations of rice shoots were significantly (one-way ANOVA, *P* < 0.05) reduced by the addition of biochar (Table 2). The Pb concentrations in the stems and leaves at the tillering stage were reduced by 79.08 and 75.64%, respectively, in soils with 5% biochar whereas the Pb concentrations in the stems, leaves, husks, and brown rice were reduced by 74.57, 81.06, 79.83, and 65%, respectively. Moreover, Pb concentration of brown rice was reduced from 0.4 mg kg^−1^ (control) to 0.14 mg kg^−1^ (5% biochar), the latter of which was below the hygienic standard for rice in China (0.2 mg kg^−1^)^26^. From statistical analysis, the Pb concentration of various parts of rice showed positive correlations with CaCl_2_-extractable Pb at the maturity stage (r_stem_ = 0.879, *P*_stem_ = 0.002; r_leaf_ = 0.913, *P*_leaf_ = 0.001; r_rice_ = 0.713, *P*_rice_ = 0.031; r_husk_ = 0.851, *P*_husk_ = 0.004, n = 9) and at the tillering stage (r_stem_ = 0.954, *P*_stem_ < 0.001; r_leaf_ = 0.842, *P*_leaf_ = 0.004, n = 9). Pb concentrations in different rice tissues were also significantly correlated with DTPA-extractable soil Pb at the maturity stage (r_stem_ = 0.671, *P*_stem_ = 0.048; r_leaf_ = 0.695, *P*_leaf_ = 0.038; r_rice_ = 0.666, *P*_rice_ = 0.050; r_husk_ = 0.672, *P*_husk_ = 0.047; n = 9), but had no correlation at the tillering stage. In line with a previous report that no significant change in metal extractability by DTPA was observed after biochar application to field soil^14^, the results indicate that CaCl_2_-extractable Pb is better than DTPA-extractable Pb at predicting the bioavailability of soil Pb in biochar treated soils.

### Effects of biochar on the transfer of Pb between different rice plant tissues

**Figure 3:**
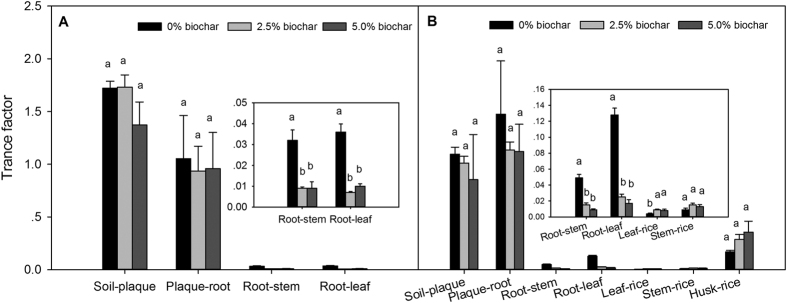
Transfer factors for Pb between different parts of the soil-rice system at tillering (**A**) and the maturity stages (**B**).

Transfer factors (TF) are used to estimate the transport of elements from soil to plants, or from one part of a plant to another^27^, and were used in this study to assess the transfer characteristics of Pb among rice tissues (Fig. 3). Biochar amendment significantly (one-way ANOVA, *P* < 0.05) decreased the root-to-leaf and root-to-stem transfer of Pb at both tillering and maturity stages. A significant reduction in TF_root-leaf_ (72.22%), and TF_root-stem_ (71.88%) occurred at the tillering stage of plants from the 5% biochar treatment, and TF_root-leaf_ and TF_root-stem_ were also reduced at the maturity stage, by 86.72 and 81.63%, respectively. However, there were no significant changes in TF_soil-iron plaque_, TF_iron plaque-root_, TF_stem-rice_, and TF_leaf-rice_ in response to biochar amendment.

Lead species in roots from the biochar treatments were determined using Pb L_3_-XANES, and the best fit was derived by incrementally increasing the number of fit components and minimizing the fit residual. The linear combination fitting (LCF) results indicated that ferrihydrite associated with Pb dominated the Pb inventory (47.1–60.8%) in roots with Fe-plaque (Table 3). Pb-pectin and Pb-cysteine complexes ranked as the second dominant forms. The roots from control treatments had more ferrihydrite associated Pb than those from biochar treatments, which could be attributed to the greater accumulation of Fe-plaque on the root surfaces of control plants, compared to plants from 5% biochar treatment group. However, with biochar application, more Pb was combined with pectin and cysteine, which can occur inside root cells. In fact, the Pb-cysteine was up to about 21.6% in the roots of the plants subjected to the 5% biochar treatment, whereas none was found in the control roots.

**Figure 4:**
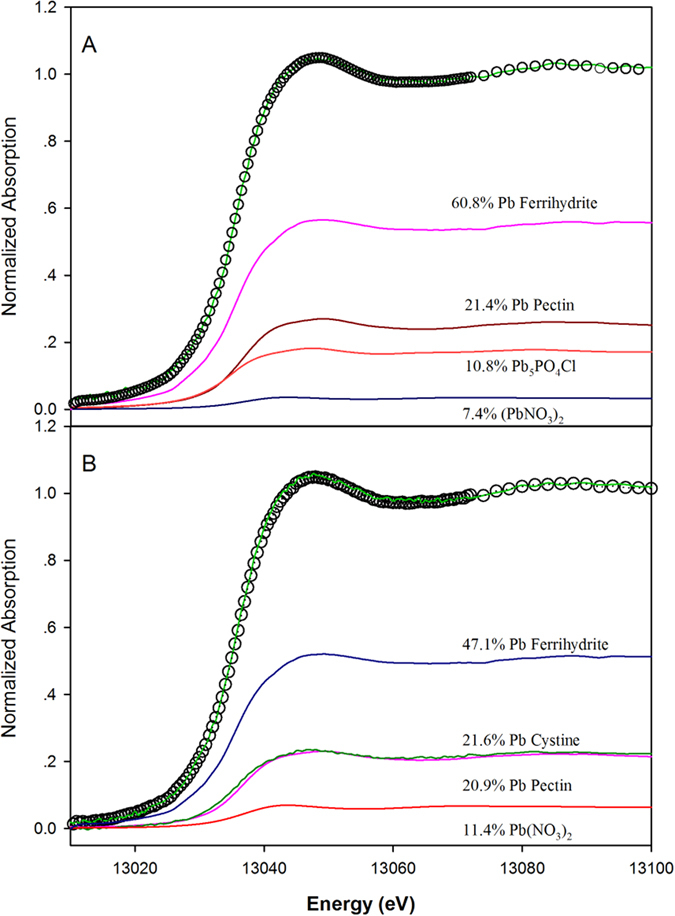
Pb L_3_ edge XANES spectra for the root samples with 0% (**A**) and 5% (**B**) biochar treatments along with the standards used to give the best fit. Solid line = measured; Open circles = best LCF fit.

There are two main processes that contributed to Pb translocation from root to shoot; (1) translocation by symplastic passage and active loading into the xylem, and (2) translocation driven by root pressure and the transpiration stream^28^. For most nonhyperaccumulating plants, Pb mainly accumulates in the roots with only a small portion being transported to shoots *via* symplastic pathways^29^,^30^. In these plants, the cell wall is the first defensive barrier that reduces Pb transfer into root cells and functions by binding Pb with polysaccharides pectins in cell walls^31^. Accordingly, the complexation of pectin with Pb^2+^ inside the root reduces the translocation of Pb from the root to the shoot^32^. Moreover, the retention capacity for Pb is linearly dependent on the number of functional groups such as -COOH, -OH, -SH, in cell walls^32^. The Pb-cysteine complex was found in the roots of the 5% biochar treatment plants (Fig. 4, Table 3). The formation of Pb-cysteine can also inhibit the translocation of Pb from the root to the shoot, resulting in relatively lower TF_root-leaf_ and TF_root-stem_ values for biochar treated rice plants.

The Pb concentration in brown rice decreased after biochar application (Table 2), and this was significantly correlated with TF_root-leaf_ (r = 0.720, *P* = 0.031, n = 9) and TF_root-stem_ (r = 0.798, *P* = 0.010, n = 9). Clearly, the decrease in Pb transfer from root to shoot, with the available soil Pb, is critical for Pb accumulation in rice grains.

## Conclusions

The amendment of biochar into a Pb-contaminated soil resulted in a decrease in soil Pb availability and amounts of Fe-plaque on root surfaces of rice. In addition, Pb concentrations in Fe-plaque were decreased after the biochar addition. Both results possibly explained the decreasing amounts of Pb uptake on rice roots. For the rice roots, we found Pb-pectin and Pb-cysteine in samples with biochar amendments. Such organic complexes could impede Pb translocation from root to shoot and subsequently lead to less Pb accumulation in brown rice with biochar addition in soils.

## Methods

### Preparation of biochar

Rice straw was collected from Minghou Agricultural Experiment Station, Fuzhou City, Fujian Province, China, and to produce biochar, the rice straw was carbonized at a pyrolysis temperature of 500 °C under anaerobic conditions in an oven for 2 h. There was also a continuous flux of nitrogen gas until the biochar had cooled down to 25 °C. The resulting biochar contained 0.37 mg kg^−1^ Cd, 6.16 mg kg^−1^ Pb, and 217.39 mg kg^−1^ Zn, and had a surface area of 18.64 m^2^ g^−1^, and a pH (H_2_O) of 10.3. The biochar was then ground, sifted through a 2-mm sieve, and stored in airtight containers prior to use as a soil amendment.

### Soil sampling and analyses

Contaminated soil was collected from the surface horizon (0–20cm) of a paddy field near a smelter (26°15′28.49″N and 118°15′12.78″E) in Youxi County, central Fujian Province, China. The soil samples were air-dried, ground, passed through a 2-mm sieve, and then analyzed (Table 4). Soil pH was determined using a pH meter (SevenCompact; Mettler-Toledo, Greifensee, Switzerland) in 5:1 water/soil suspensions. Cation-exchange capacity (CEC) was determined using 1 M ammonium acetate buffered at pH 7.0. Soil organic matter was determined using the K_2_Cr_2_O_7_ wet oxidation method. Soil particle-size distribution (<2 mm fractions) was determined using the pipette method after hydrogen peroxide treatments^33^.

### Growth conditions and seed treatment

All of our pot experiments were conducted in a greenhouse at the College of Resource and Environmental Sciences, Fujian Agriculture and Forestry University, Fuzhou City, Fujian Province, South-eastern China. Rice seeds (Donglian 5 of conventional *Oryza sativa* L. *ssp. indica*) were sterilized in a solution containing 1% sodium hypochlorite solution for 15–30 min and germinated in dishes containing moist tissue paper for 3 d. After germination, rice seedlings were selected transferred to a beaker, and grown for 12 d. Subsequently, five homogeneous seedlings were selected transplanted into each pot, and the potted plants were grown in a greenhouse under normal sunlight.

The pots used in this study were 9L ceramic pots that measured 20 cm (bottom) and 28 cm (top) in diameter and 17 cm in height and they will filled with 7.5 kg air-dried soil, 2.1 g urea, 1.2 g NH_4_H_2_PO_4_, and 2.1 g K_2_SO_4_. In addition, the soil mixtures were amended with 0% (control, absence of biochar), 2.5%, or 5% biochar by weight. The mixtures were equilibrated 10 d at 70% of water holding capacity. All experiments were performed in triplicates. Each pot was submerged in water, and the water level was maintained at approximately 1–2 cm above the soil surface during the entire growing period.

### Sample collection and analysis

Soil samples and rice plants were collected from each pot at the tillering and maturity stages. The soil samples were air-dried, ground with a wood roller, and passed through a 2-mm sieve. A small portion of the soil was further ground with an agate mortar to pass a 0.149-mm sieve and was subsequently used for chemical and physical analyses.

Bioavailable Pb in the soils was extracted using a CaCl_2_ solution (0.1 M) and a DTPA solution (diethylene triamine pentaacetic acid, 0.005 M; CaCl_2,_ 0.01 M; triethanolamine [TEA], 0.1 M; pH = 7.3), according to the following procedures: 5.00g air-dried soil (<2 mm) was mixed with 25 mL of CaCl_2_ solution or DTPA solution. The soil suspension was continuous shaken at 25 °C for 2 h and then immediately filtered. Exactly 1.0 g of the air-dried soil was then mixed with 25 mL decarbonized distilled deionized water (DDW) and shaken at 25 °C for 2 h. Then the soil suspension was filtered through a Millipore filter (0.45 μm), and the filtrated was used for water-soluble organic carbon (WSOC) analysis, which was detected using a TOC analyzer (model 5000A; Shimadzu, Tokyo, Japan).

Plant shoot tissues were separated into stems, leaves, husks, and brown rice. The plant tissues were deep-washed in DDW, and after drying at 70 °C until constant weight, the dry weights of the aerial portions of the plants in each pot were recorded.

Iron plaque was extracted from fresh root surfaces using a dithionite-citrate-bicarbonate (DCB) solution, containing 0.03 M sodium citrate (Na_3_C_6_H_5_O_7_·2H_2_O), 0.125 M sodium bicarbonate (NaHCO_3_), and 0.06 M sodium dithionite (Na_2_S_2_O_4_). The rice roots were dipped into the DCB solution (40 mL) at 25 °C for 60 min, and then rinsed three times with DDW. The water used for rinsing was mixed with the DCB extracts for a total volume of up to 100 mL, and the concentrations of Pb and Fe in the diluted DCB extracts were measured. After extraction with the DCB solution, fresh roots were oven dried for 48 h at 70 °C for further analysis.

The dried plant tissues were pulverized into a powder and 0.1–0.2 g of the powder was digested with HNO_3_/hyperchloric acid in heating blocks^34^. After digestion, the sample solutions were dried and dissolved in 0.5 M HNO_3_, filtered through a Millipore filter (0.45 μm), then stored in plastic bottles for subsequent analysis. The Pb concentration was measured using an Induced Couple Plasma-Mass Spectrometer (ICP-MS, NexION 300X; Perkin Elmer, NY).

### Sequential extraction of soil Pb

After harvest, soils with and without biochar were subjected to a European Community Bureau of Reference (BCR) sequential extraction, using a three-stage modified procedure recommended by the BCR methodology^35^. Briefly, four metal fractions, including exchangeable and acid soluble (F1), reducible (F2), oxidizable (F3), and residual (F4) fractions, were determined (see supporting information for details).

### Lead L_3_-edge X-ray absorption spectroscopy (XAS)

The collected root samples were freeze drying for three days, pulverized into powders, and passed through a 0.149-mm mesh sieve. Approximately 0.2 g of the root samples were mounted in acrylic holders, sealed with Kapton tape to avoid desiccation, and stored at 4 °C until analysis. Spectra were collected at room temperature using the Beamline BL12B at Spring-8, Hyogo Prefecture, Japan, where the storage ring was operated at a fixed current of 100 mA. The Si (111) monochromator energy was calibrated to 13035 eV based on the first inflection point in the L_3_-edge derivative spectra from a Pb foil. Spectra were collected in fluorescence or transmission mode with a Lytle detector or gas ionization chamber at photon energies between −200 to +750 eV relative to the PbL_3_-edge energy at 13,035 eV, using a step size of 0.5 eV across the absorption edge region (−30 to +40 eV), and a step size of k = 0.06 Å^−1^ at higher energies.

At least three scans were acquired for each sample. Multiple XAS scans were aligned, merged and processed using the Athena program, an interface to IFEFFIT^36^,^37^ The backgrounds of the spectra were corrected using a linear pre-edge function between −200 and −30 eV and a linear or quadratic function between 55 and 400 eV, included a flattening function in the post-edge region for normalization. The distributions of the Pb species in the samples were determined using LCF across the region from 30 eV below to 33 eV above the PbL_3_ edge.

Reference materials for LCF included Pb complexed with biochar and adsorbed on kaolinite, ferrihydrite, Al-hydroxide, birnessite, humic acid, and root cell walls, which were prepared by mixing 250 mL of 0.5 mM Pb(NO_3_)_2_ with 2.5 g of sorbents and incubating the suspensions at pH 5 for 48 h. In addition, the organic Pb complexes of Pb-malate, Pb-citrate, Pb-cysteine, Pb-oxalate, Pb-pectin, Pb-acetate, and Pb-glutathione were prepared by adding 12.5 mL of 100.0 mM malate, citrate, cysteine, oxalate, pectin, acetate, glutathione, respectively, to 1.25 mL of 100 mM Pb(NO_3_)_2_, diluting them with 0.5 mM HNO_3_ to 250 mL and then mixing them at pH 5 for 24 h. The references of PbS, Pb(NO_3_)_2_, PbCl_2_, PbO, PbO_2_, PbSO_4_, Pb_5_(PO_4_)_3_Cl, and Pb(OH)_2_·2PbCO_3_ were purchased from Sigma-Aldrich whereas Pb_3_(PO_4_)_2_ was synthesized as described by Wruck^38^.

### Calculation of the transfer factor

The transfer factor from part “a” to part “b” was calculated using the following formula:


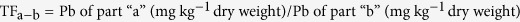


### Data processing and statistics

One-way analysis of variance (ANOVA) and the Duncan multiple range test (DMRT) were used to test for significant differences between the Pb concentrations of different plant tissues and soils, according to a 5% level of significance. The correlation coefficient was computed using SPSS, version 18.0 (SPSS Institute, Chicago, IL, 2009).

## Additional Information

**How to cite this article**: Li, H. *et al*. Biochar amendment immobilizes lead in rice paddy soils and reduces its phytoavailability. *Sci. Rep.* **6**, 31616; doi: 10.1038/srep31616 (2016).

## Supplementary Material

Supplementary Information

## Figures and Tables

**Table 1:** CaCl_2_-and DTPA-extractable Pb in soil amended with biochar (mean ± S.E., n = 3).

Biochar added	Tillering stage			pH	Maturity stage			pH
	CaCl_2_-Pb (mg kg^−1^)	DTPA-Pb (mg kg^−1^)	WSOC (mg kg^−1^)		CaCl_2_-Pb (mg kg^−1^)	DTPA-Pb (mg kg^−1^)	WSOC (mg kg^−1^)	
0%	10.04 ± 1.54a^a^	616.7 ± 19.76ab	321.22 ± 20.70b	5.63 ± 0.24c	35.29 ± 5.49a	750.2 ± 13.39a	180.79 ± 10.63b	5.31 ± 0.27b
2.50%	3.30 ± 0.46b	639.8 ± 14.49a	426.26 ± 16.31ab	6.09 ± 0.06b	12.68 ± 0.83b	709.1 ± 26.82a	166.45 ± 4.28b	5.48 ± 0.10b
5%	0.33 ± 0.24b	508.9 ± 46.02b	599.85 ± 68.03a	6.81 ± 0.31a	2.00 ± 0.16b	625.4 ± 16.87b	270.57 ± 12.02a	6.11 ± 0.13a

^*a^The same letter in a column indicates no significant difference at *P* < 0.05 according to Duncan’s multiple range test.

**Table 2:** Lead concentration in different parts of the rice plant (mg kg^−1^, mean ± SE, n = 3).

Biochar added	Tillering stage				Maturity stage				Husk	Brown rice
	Root (without iron plaque)	Root (with iron plaque)	Stem	Leaf	Root (without iron plaque)	Root (with iron plaque)	Stem	Leaf		
0%	707.1 ± 42.17a^a^	2176 ± 423.9a	21.56 ± 1.21a	22.78 ± 2.60a	870.1 ± 77.42b	1553 ± 62.17a	41.73 ± 2.09a	111.6 ± 14.90a	2.33 ± 0.39a	0.40 ± 0.10a
2.50%	849.2 ± 153.1a	2104 ± 65.45a	8.73 ± 0.68b	6.69 ± 1.01b	918.9 ± 95.43ab	1543 ± 50.68a	13.31 ± 0.92b	22.67 ± 3.78b	0.72 ± 0.13b	0.20 ± 0.03ab
5%	570.7 ± 132.9a	1278 ± 26.67b	4.51 ± 0.62c	5.55 ± 1.20b	1194 ± 83.26a	1722 ± 401.9a	10.61 ± 0.60b	21.14 ± 6.22b	0.47 ± 0.16b	0.14 ± 0.02b

^*a^The same letter in a column indicates no significant difference at *P* < 0.05 according to Duncan’s multiple range test.

**Table 3:** The results of linear combination fitting for the Pb L_3_-edge XANES spectra of roots (with iron plaque).

Biochar added	Weighted percentage (%) of the roots					
	Pb(NO_3_)_2_	Ferrihydrite	Pectin	Cysteine	Pb_5_PO_4_Cl	***R-factor*** (**×**10^3^)^b^
0%	7.4 ± 1.6	60.8 ± 3.4	21.4 ± 2.1	0	10.8 ± 2.5	0.21
5%	11.4 ± 1.0	47.1 ± 1.7	20.9 ± 1.9	21.6 ± 3.1	0	0.12

The data show the proportion (%) of the reference spectra that resulted in the best fit to the sample data^a^.

^a^Mean ± standard deviation. Weighting factors on each fit summed to 100 ± 3%.

^b^Normalized sum of the squared residuals of the fit (*R-factor* = ∑(data-fit)^2^/∑data^2^).

**Table 4:** Physicochemical properties of the Pb-contaminated soil used in this study.

Parameter	Value
pH	6.05
CEC (cmol^+^ kg^−1^)	17
Organic matter (g kg^−1^)	21.86
Metals (mg kg^−1^)	
Cd	5.88
Pb	1602
Zn	2132
Exchangeable cation (cmol^+^ kg^−1^)	
Ca	6.94
Mg	1.06
K	0.84
Na	0.51
Soil texture	Silty loam
Particle size distribution (%)	
Sand	44.56
Silt	48.21
Clay	7.23
