# Supplementary material for: Biochar amendment immobilizes lead in rice paddy soils and reduces its phytoavailability
Source: Sci Rep. 2016 Aug 17;6:31616. doi: 10.1038/srep31616 (PMC4987687; doi:10.1038/srep31616)
Supplement: Supplementary Information [file srep31616-s1.doc]

**Title: Biochar amendment immobilizes lead in rice paddy soils and reduces its phytoavailability**

**Authors**: Honghong Li^1^, Yuting Liu^2^, Yanhui Chen^1^, Shanli Wang^3^, Mingkuang Wang^1,3^, Tuanhui Xie^1^, Guo Wang^1^,*

**Affiliations**:

^1^ College of Resource and Environmental science, Fujian Agriculture and Forestry University, Fuzhou 350002, PR China

^2^ Department of Soil and Environmental Sciences, National Chung-Hsing University, Taichung, Taiwan, 10402

^3^ Department of Agricultural Chemistry, National Taiwan University, Taipei, Taiwan, 10617

**Supplemental Information:**

**The DCB procedure**

For each soil extraction, 1.0 g air-dried soil was weighed to three decimal places and added to a 100 mL polyethylene centrifuge tube. Four fractions were determined for each metal: exchangeable and acid soluble (F1), reducible (F2), oxidizable (F3) and residual (F4) fractions. All data reported were the averaged values of three measurements (n=3). A soil sample with certified concentrations of Pb (GBW080037) was provided by the China National Center for Standard Materials and was used as a reference for quality control.

Step 1 (exchangeable/acid extractable fraction): A total of 40 mL acetic acid solution (0.11 mol L^-1^, pH = 3.06) was added to the centrifuge tube and shaken for 16 h at 25^o^C. The extract was separated from the solid residue by centrifugation at 3,000 x g for 20 min. The supernatant was collected for later analysis. The residue was rinsed with 20 mL DDW by shaking for 15 min, centrifuging and decanting the supernatant.

Step 2 (easily reducible fraction): A total of 40 mL freshly prepared hydroxylamine hydrochloride solution (0.5 mol L^-1^, pH = 1.60) was added to the residue in the centrifuge tube from step 1 and shaken for 16 h at 25^o^C. The separation of the extract, collection of the supernatant, and rinsing of residue were the same as in step 1.

Step 3 (oxidizable fraction): First, 10 mL of hydrogen peroxide (30%) was added to the residue from step 2 in the centrifuge tube. The centrifuge tubes were loosely covered with a lid to prevent substantial loss of hydrogen peroxide. The digestion at 25^o^C was allowed to proceed for 1 h with occasional manual shaking and this was then followed by digestion at 85^o^C for another 1 h. The centrifuge tubes were uncovered and heating continued to reduce the volume to less than 3 mL. Another 10 mL hydrogen peroxide was added to the centrifuge tube and the contents digested with a cover at 85^o^C for 1 h. And again, the lid was removed and the volume reduced to 3 mL. Finally, 50 mL of 1.0 mol L^-1^ ammonium acetate solution was added to the contents were shaken for 16 h at 25^o^C. The separation of the extract, collection of the supernatant, and rinsing of the residues were the same as described in step 1.

Step 4 (residual fraction): Residue from step 3 was heated in a water bath until dry at 60^o^C. The dried soil was transferred to a bottle and then digested by HCl-HNO_3_-HF-HClO_4_ for determination of the residual Pb concentration.

.

**Figure S1 (Pb standard XANES spectra)**


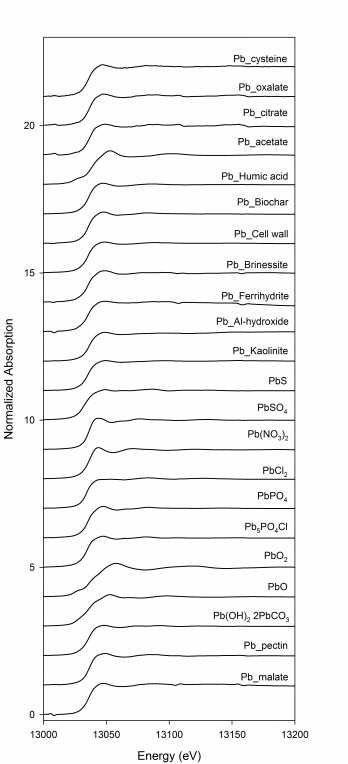


**Fig. S1. Pb L-Ⅲ edge XANES spectra for the reference standards**
